# Decrease of gene expression diversity during domestication of animals and plants

**DOI:** 10.1186/s12862-018-1340-9

**Published:** 2019-01-11

**Authors:** Wei Liu, Lei Chen, Shilai Zhang, Fengyi Hu, Zheng Wang, Jun Lyu, Bao Wang, Hui Xiang, Ruoping Zhao, Zhixi Tian, Song Ge, Wen Wang

**Affiliations:** 1 State Key Laboratory of Genetic Resources and Evolution, Kunming Institute of Zoology, Chinese Academy of Sciences, Kunming, 650223 China; 2 Kunming College of Life Science, University of Chinese Academy of Sciences, Kunming, 650204 China; 3 State Key Laboratory of Systematic and Evolutionary Botany, Institute of Botany, Chinese Academy of Sciences, Beijing, 100093 China; 4 State Key Laboratory of Plant Cell and Chromosome Engineering, Institute of Genetics and Developmental Biology, Chinese Academy of Sciences, Beijing, 100101 China; 5 School of Agriculture, Yunnan University, Kunming, 650091 Yunnan China; 6 Center for Ecological and Environmental Sciences, Key Laboratory for Space Bioscience & Biotechnology, Northwestern Poly-technical University, Xi’an, 710072 China; 7 Guangzhou Key Laboratory of Insect Development Regulation and Application Research, Institute of Insect Science and Technology and School of Life Sciences, South China Normal University, Guangzhou, 510631 China

**Keywords:** Domestication, Decrease, Gene expression diversity

## Abstract

**Background:**

The genetic mechanisms underlying the domestication of animals and plants have been of great interest to biologists since Darwin. To date, little is known about the global pattern of gene expression changes during domestication.

**Results:**

We generated and collected transcriptome data for seven pairs of domestic animals and plants including dog, silkworm, chicken, rice, cotton, soybean and maize and their wild progenitors and compared the expression profiles between the domestic and wild species. Intriguingly, although the number of expressed genes varied little, the domestic species generally exhibited lower gene expression diversity than did the wild species, and this lower diversity was observed for both domestic plants and different kinds of domestic animals including insect, bird and mammal in the whole-genome gene set (WGGS), candidate selected gene set (CSGS) and non-CSGS, with CSGS exhibiting a higher degree of decreased expression diversity. Moreover, different from previous reports which found 2 to 4% of genes were selected by human, we identified 6892 candidate selected genes accounting for 7.57% of the whole-genome genes in rice and revealed that fewer than 8% of the whole-genome genes had been affected by domestication.

**Conclusions:**

Our results showed that domestication affected the pattern of variation in gene expression throughout the genome and generally decreased the expression diversity across species, and this decrease may have been associated with decreased genetic diversity. This pattern might have profound effects on the phenotypic and physiological changes of domestic animals and plants and provide insights into the genetic mechanisms at the transcriptome level other than decreased genetic diversity and increased linkage disequilibrium underpinning artificial selection.

**Electronic supplementary material:**

The online version of this article (10.1186/s12862-018-1340-9) contains supplementary material, which is available to authorized users.

## Background

Domestic species usually undergo dramatic phenotypic and physiological changes in response to strong artificial selection [1, 2], usually show lower adaptability to their original harsh wild environments and even acquire “domestication syndrome” [3–5], such as loss of dormancy, loss of seed shattering [6, 7], and increased fruit or grain size [8] in plants and less aggression, reduced fear of humans, changed coat colour, reductions in tooth size, and alterations in ear and tail form in animals [5, 9]. Despite thousands of years of agricultural practices and 150 years of scientific research since Darwin [1, 2], much effort is still necessary to reveal the general genetic basis underlying the domestication of animals and plants. In recent years several plant domestication genes have been identified, such as *sh4*, which reduced seed shattering in cultivated rice [6]; *PROG1*, which affected tiller angle and the number of tillers in rice [10]; and *fw2.2*, which increased fruit size in domesticated tomato [8]. Therefore, it has been postulated that mutations in a few loci might have contributed to major domestication traits [11, 12]. Genome-wide scans for signatures of artificial selection further indicated that a small percentage of genes were affected during domestication, such as 2~4% of genes in maize [13] and 6.67% of genes in soybean [14], and revealed that domestic species usually showed decreased genetic diversity [13, 15, 16] and increased linkage disequilibrium [14, 17–19] compared with its wild relatives.

Although only a small percentage of genes might have been involved in domestication, well-domesticated species usually show extensive phenotypic and physiological changes that make them substantially different from their wild ancestors. Some studies have revealed that different genetic variations, including single nucleotide variants in both coding and regulatory regions, copy number variations, insertions and deletions, could explain the morphological changes [12, 16, 20]. Conceivably, some of these genetic variations may result in morphological changes through changing the expression of genes. Therefore, the transcriptome, which is the connection between genotypes and phenotypes, might play a role during domestication [16]. Recent high-throughput sequencing technologies have made it possible to focus on genome-wide expression changes, and several studies have been conducted to find genome-wide expression differences during domestication by comparing the transcriptomes of domestic and wild species [21–24]. However, all these previous comparative transcriptomic studies focused on differentially expressed genes (DEGs) between domestic and wild species, usually restricted in one species. Therefore, it is worth investigating whether or not domestic plants and animals show patterns at the transcriptome level similar to the decreased genetic diversity and increased linkage disequilibrium observed at the genomic level.

In this study, we systematically generated and collected transcriptome data for three domestic animals, four cultivated plants and their corresponding wild progenitors, i.e., from a total of seven representative domestic-wild pairs. Interestingly, the gene expression diversity levels tend to be lower in domestic species than in corresponding wild species, and this decrease may be an important pattern related to expression level and may be the result of artificial selection for specific traits under domestication or for survival in the suitable environments associated with care provided by humans. In other words, domestication might have been a process in which some unnecessary variation in genetic expression was discarded to give rise to the traits that humans selected, fitting a “less is more” mode [25] and in extreme cases, leading to domestication syndrome [26].

## Results

### Transcriptome data

We sequenced the mRNA extracted from the panicles of 20 wild rice accessions (*Oryza rufipogon* and *Oryza nivara*) and 20 cultivated rice (*Oryza sativa*) accessions (including the *indica*, *aus*, *aromatic*, *temperate japonica* and *tropical japonica* cultivar groups) [27] (Additional file 1: Table S1**)**, the stem apical meristems of 35 soybean samples (Additional file 1: Table S2) including 10 wild soybean accessions (*Glycine soja*), 14 landraces and 11 improved cultivars and the silk glands of silkworms including 4 wild individuals (*Bombyx mandarina*) and 4 domestic accessions (trimolter silkworms of *B. mori*) (Additional file 1: Table S3). Sequencing yielded a total of 1.38 billion high-quality cleaned paired-end reads for rice, which were 100 bp in length (Additional file 1: Table S4); 0.87 billion reads for soybeans, which were 100 bp in length (Additional file 1: Table S5); and 0.22 billion reads for silkworms, which were 121 bp in length (Additional file 1: Table S6). We also collected transcriptome data from other four domestic species for which transcriptome data were available for both domestic species and their wild progenitors, including the brain frontal cortexes of dog and wolf [22], gastrocnemius of domestic and wild chicken [21], leaf of cultivated and wild cotton [28] and ear, stem and leaf of maize and teosinte [29]. Consequently, a total of seven pair-wise statistically sufficient transcriptome datasets (more than 4 replicates for each tissue type) for both the domestic species and corresponding wild progenitors were used for the following analysis (Table 1).

**Table 1:** Summary of all the transcriptome data

Species	Type	Breed	Sample size	Tissue	Data sources
Rice	Dome	*Oryza sativa indica*	9	panicle	This study
	Dome	*Oryza sativa japonica*	11	panicle	
	Wild	*Oryza nivara*	10	panicle	
	Wild	*Oryza rufipogon*	10	panicle	
Soybean	Improved	*Glycine max*	11	stem apical meristems	This study
	Landrace	*Glycine max*	14	stem apical meristems	
	Wild	*Glycine soja*	10	stem apical meristems	
Maize ear	Dome	Maize	12	ear	[29]
	Wild	Teosinte	18	ear	
Maize leaf	Dome	Maize	12	leaf	
	Wild	Teosinte	17	leaf	
Maize stem	Dome	Maize	12	stem	
	Wild	Teosinte	17	stem	
Cotton	Dome	*Gossypium hirsutum*	40	leaf	[28]
	Wild		10	leaf	
Silkworm	Dome	*Bombyx mori* (trimolter)	4	silk gland	This study
	Wild	*Bombyx mandarina*	4	silk gland	
Dog	Dome	Dog	5	brain frontal cortexes	[22]
	Wild	Grey wolf	6	brain frontal cortexes	
Chicken	Dome	Avian broiler	5	gastrocnemius	[21]
	Wild	Red junglefowl	4	gastrocnemius	

Dome represents the domestic species, while wild represents the wild progenitor species. Panicles of rice samples, stem apical meristems of soybeans and silk glands of silkworms were sequenced by us. The domestic silkworm samples were from four trimolter silkworm breeds (Additional file 1: Table S3). A few of the samples which had lower mapping depths were discarded in the following analysis (Additional file 2: Table S8)

Among the seven pairs, data from the panicles of rice pairs, stem apical meristems of soybean pairs and silk glands of silkworm pairs, which were generated in this study, had higher average mapping depths in exonic regions, equaling 68×, 34× and 104×, respectively. The average mapping depth for cotton pairs was approximately 42×, and that for the brain frontal cortex of dog and wolf both was approximately 16×. The ear, leaf and stem of maize and teosinte had an approximately 10× average mapping depth. Although the average mapping depths differed among the seven pairs, the average mapping depths were very similar between each domestic species and its corresponding wild species (Additional file 1: Table S7 and Additional file 2: Table S8).

We also measured the expression level of all the genes of each pair with fragments per kilobases per million mapped reads (FPKMs) values. When the FPKM value is greater than 1, the gene is considered an expressed gene [23]. The number of expressed genes was not significantly different between the domestic species and their wild progenitors (Additional file 1: Table S7), suggesting that the number of expressed genes changed little during domestication. Other FPKM thresholds, such as 0, 0.1, 0.5, and 5, were also used to count the number of expressed genes and the conclusions remained the same as those for a threshold of 1 (Additional file 1: Table S7).

### Variation of gene expression diversity

Regular transcriptome analysis focuses more on DEGs [21–24], but little is known about the global change of gene expression pattern during domestication. Here, we calculated the gene expression diversity, which represents the gene expression variation levels in a transcriptome and is measured by the coefficient of variation (CV) in gene expression [30], separately for the wild and domestic species.

**Fig. 1:**
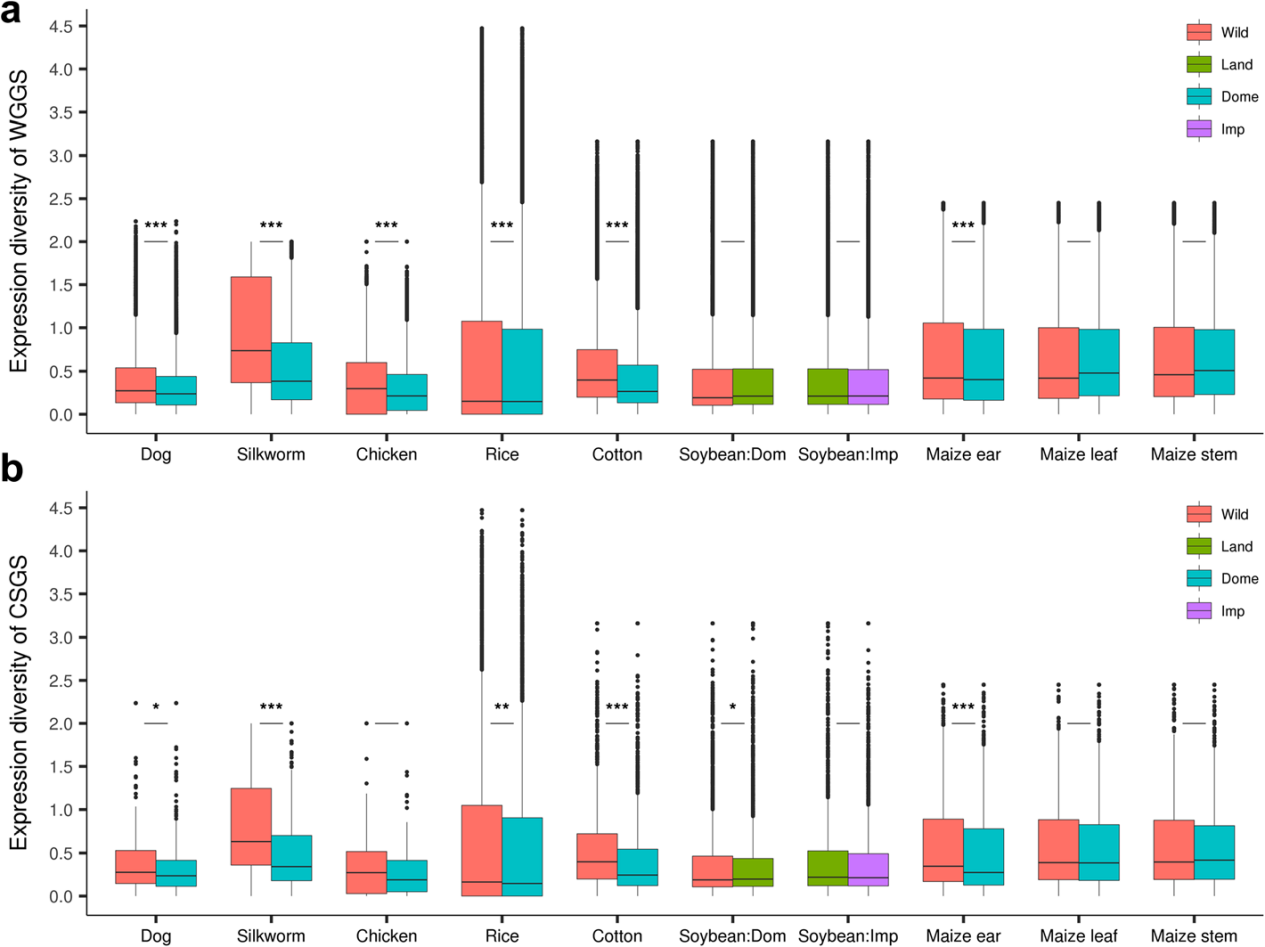
Gene expression diversity in the whole-genome gene set (WGGS) and candidate selected gene set (CSGS) for the seven pairs. **a** Expression diversity of the WGGS. **b** Expression diversity of the CSGS. The samples of soybean could be clearly classified as wild, landraces and improved cultivars. The other six pairs were grouped into wild and domestic species. The markers above the solid black lines are the *P*-value from a Student’s *t*-test of whether the expression diversity values in the domestic species are significantly lower than those in the wild species and the *P*-value less than 0.05, 0.01 and 0.001 are marked with *, ** and ***, separately. The expression diversity changes of the two subgenomes of cotton can be found in the supplementary information (Additional file 1: Figure S1)

Interestingly, the expression diversity values for the whole-genome gene set (WGGS) of the domestic species were generally lower than those of the corresponding wild species. Five of seven domestic species consistently showed significantly lower expression diversity than the wild species in the WGGS based on Student’s *t*-test (Fig. 1a, Table 2), including dog (10.2% decrease, *P* < 2.2e-16), silkworm (37.7% decrease, *P* < 2.2e-16), chicken (14.2% decrease, *P* < 2.2e-16), rice (5.1% decrease, *P* = 1.072e-12) and cotton, for which both the whole-genome genes and the two-subgenome genes showed decreased expression diversity (whole genome,16.4% decrease, *P* < 2.2e-16; A subgenome (At), 15.9% decrease, *P* < 2.2e-16; D subgenome (Dt), 17. 1% decrease, *P* < 2.2e-16) (Additional file 1: Figure S1a). The leaf gene expression diversity of maize was not significantly lower than that of teosinte (0.691 in maize vs 0.684 in teosinte, *P* = 0.92), and the stem gene expression diversity of maize was almost the same as that of teosinte (0.696 in maize vs 0.697 in teosinte). However, the ear of maize showed significantly lower expression diversity than that of its wild related species (5.1% decrease, 0.660 in maize vs 0.696 in teosinte, *P* < 8.776e-14) (Fig. 1a, Table 2). For soybean, the gene expression diversity of landraces (0.487) and improved cultivars (0.482) were very similar to that of the wild species (0.485) (Fig. 1a, Table 2). Given the fact that the soybean landraces and improved cultivars sampled in this study experienced similar depletion of genetic diversity to other domestic species (Additional file 1: Table S12), it is unknown why soybean didn’t show decreased gene expression diversity during domestication. One explanation is that soybean may experience unique diverse selection, as indicated by different traits of stem, leaf and photoperiod sensitivity in different landrace and cultivar groups [14]. In this study, our samples were from different distinct groups (Additional file 1: Table S2). To initially test this hypothesis, we randomly chose four samples from a single group landraces and four wild soybean accessions to calculate gene expression diversity, and found that the four landraces indeed showed significantly decreased expression diversity (2.5% decrease, *P* = 2.1 × 10^− 3^) (Additional file 1: Figure S2a), indicating specific genetic background may also function in the decrease of gene expression diversity in soybean although the effect may not be as strong as in other domestic species. In addition to the domestic species in this study, previously reported data showed that the gene expression diversity of the common bean is 18% lower than that of its wild related species [30]. Altogether, these results indicate that domestic animals and plants tend to lose expression diversity during domestication.

**Table 2:** Gene expression diversity changes in the seven domestic and wild species

Species	Pair	All		Candidate selected genes			Non-CSGS
		WGGS	Dcv of WGGS	CSGS	PCSGS	Dcv of CSGS	Dcv of non-CSGS
Dog	Dog-Wolf	24,580	10.2%***	294	1.20%	16.1%*	10.2%***
Silkworm	Trimolter-Wild	15,665	37.7%***	421	2.69%	34.0%***	37.8%***
Chicken	AB-RJF	17,858	14.2%***	148	0.83%	19.1%	14.1%***
Rice (*japoniaca*)	Dome-Wild	91,080	5.1%***	6892	7.57%	7.0%**	5.0%***
Rice (*nivara*)	Dome-Wild	37,985	12.5%***				
Soybean	Landrace-Wild	54,174	−0.4%	3614	6.67%	5.6%*	−0.8%
	Improved-Landrace		1.1%	2987	5.51%	4.3%	0.8%
Cotton	Dome-Wild	70,478	16.4%***	1777	2.52%	20.6%***	16.3%***
Cotton.At	Dome-Wild	32,032	15.9%***	549	1.71%	17.2%**	15.8%***
Cotton.Dt	Dome-Wild	34,402	17.1%***	1228	3.57%	21.9%***	16.9%***
Ear of maize	Maize-Teosinte	39,621	5.1%***	1606	4.05%	13.0%***	4.9%***
Leaf of maize	Maize-Teosinte		−1.0%			5.6%	−1.3%
Stem of maize	Maize-Teosinte		0.2%			4.4%	0%

WGGS: Number of genes in the whole genome gene set; D_cv_ of WGGS: Decreased percentages of the expression diversity for the WGGS; CSGS: Number of genes in candidate selected gene set; PCSGS: Percentage of the number of candidate selected genes; D_cv_ of CSGS: Decreased percentages of expression diversity for the CSGS; Non-CSGS: Number of non-candidate selected genes; and D_cv_ of non-CSGS: Decreased percentage of expression diversity for non-CSGS; the species whose *P*-value less than 0.05, 0.01 and 0.001 are marked with *, ** and ***, respectively. The decreased percentage of expression diversity (D_cv_) is equal to 1-(CV_dome_-CV_wild_) and the expression diversity is represented by the coefficient of variation (CV) in expression level. For rice, genes in the CSGS are from our analyzed data (Additional file 2: Table S10), and the candidate selected genes of the other six pairs are based on previously published data [14, 18, 28, 31, 32, 34, 35]. Detail information about the expression diversity of the seven pairs can be found in the supplemental table (Additional file 2: Table S11)

Because the genomes of domestic species (except for chicken) were used as the reference genomes for mapping, and the wild species usually have lower read mapping ratios compared to the domestic species (Additional file 1: Figure S3), it is necessary to determine whether mapping bias caused by genetic differences between the genomes of domestic and wild species would reverse the pattern of decreased expression diversity. To test this, we mapped the reads of rice by using the reference genome of the wild species, *O. nivara* (GCA_000576065.1), and analysed the gene expression diversity of the wild and cultivated rice. The degree of decreased gene expression diversity of the cultivated species (1.054) compared to the wild species (1.205) was even higher (12.5% decrease, *P* < 2.2e-16) than that obtained using the genome of *Oryza japonica* as the reference (5.1% decrease, *P* < 1.1e-12) (Table 2, Additional file 1: Figure S4), indicating that a lower mapping ratio may underestimate the expression diversity of wild species and the degree of decreased expression diversity when the genome of domestic species is used. In addition, we also observed significantly lower expression diversity in the domestic chicken when using the genome of wild chicken (*Gallus gallus*) as the reference genome (Fig. 1a, Table 2). These results suggest that mapping ratio differences caused by reference genome difference between the domestic and wild species do not change the observed result.

### Expression diversity in the candidate selected gene set

We further investigated the changes of gene expression diversity in the candidate artificially selected genes. For the seven pairs, the candidate regions that underwent selective sweeps during domestication have been previously reported [14, 18, 28, 31–35]. We put the genes located in the candidate selective sweep regions into the candidate selected gene set (CSGS) for each domesticated species and the other genes not located in these selective sweep regions were placed in the non-candidate selected gene set (non-CSGS).

For rice, a well-known previous study identified 10,674 candidate selected genes, which represented 11.72% of the whole genome genes [33]. Perhaps due to the lower sequencing depth used at that time, the selective sweeps identified in rice in that study may not be accurate because the percentage of candidate selected genes is much larger in rice than in the other species: 7.3% in sunflower [36], 4.05% in maize [13, 34] and 6.67% in soybean [14] (Table 2). Therefore, we used 144 samples (Additional file 2: Table S9) which included 42 wild rice accessions from the NCBI (PRJEB2829) and 102 cultivated accessions from the 3000 Rice Genomes Project [37] to reanalyse the selective sweeps in rice. Finally, we identified 95 selective sweep regions using a.

likelihood method (XP-CLR). These regions contained only 6892 candidate selected genes and represented 7.57% of the whole-genome genes (Table 2, Additional file 2: Table S10). Several well-characterized domesticated genes were contained in the new candidate selected gene list, including *An-1* [38] (awn development), *An-2* [39] (*LOGL6*, awn length regulation), *GAD1* [40] (grain development), *OsC1* [41] (leaf sheath colour and apiculus colour), *OsLG1* [42] (panicle architecture), *sh4* [6] (seed shattering), and *PROG1* [10] (*PROSTRATE GROWTH 1*, tiller angle and number of tillers), indicating that rice candidate selected regions were well identified in our new results (Additional file 1: Figure S5). Therefore, fewer than 8% of the whole-genome genes were affected during domestication in different representative domestic species (Table 2).

After obtaining the CSGS (Table 2) for each domestic species, we calculated the expression diversity for the CSGS and non-CSGS. For the CSGS, pair-wise comparisons between domestic and wild species of dog, silkworm, rice, cotton, landrace soybean and maize (ear) revealed significantly (*P* < 0.05) lower expression diversity in the domestic species. In addition, both subgenomes of cotton, namely, the At (17.2% decrease) and Dt (21.9% decrease) subgenomes (Table 2, Additional file 1: Figure S1b, Additional file 2: Table S11), had significantly lower expression diversity in the domestic species for the CSGS. Unlike in the WGGS, the landraces of soybean showed significantly decreased expression diversity in the candidate domesticated gene set (5.6% decrease, *P* = 0.046) (Fig. 1b, Table 2). Except the gene expression diversity of CSGS for chicken (*P* = 0.071), the leaf (*P* = 0.054) and stem (*P* = 0.087) of maize, and the improved soybean (*P* < 0.1146) were not significant, all the domestic species showed various degrees of decreased expression diversity in the CSGS, and the percentages reduction in expression for dog, silkworm, chicken, rice, landrace and improved soybean, cotton, and the ear, leaf and stem of maize were 16.1, 34.0, 19.1, 7.0, 5.6, 4.3, 20.6, 13.0, 5.6 and 4.4%, respectively (Table 2).

To examine whether the general decrease of gene expression diversity in the WGGS was caused solely by the selected gene set, we also investigated the gene expression diversity in the non-CSGS. Intriguingly, the non-CSGS also generally showed lower expression diversity in domestic species than in their corresponding wild counterparts (except in soybean and in the leaf of maize) (Additional file 1: Figure S6), although the degree of decrease was weaker than that for the CSGS, with only a single exception in the silkworm (Table 2, Additional file 2: Table S11). These results suggested that the CSGS contributed more to the decreased expression diversity of the WGGS than did the non-CSGS. Moreover, for the two subgenomes of cotton, the Dt exhibited a higher degree of decreased expression diversity than did the At in both the WGGS (17.0% decrease in Dt vs 15.9% decrease in At) and CSGS (21.9% decrease in Dt vs 17.2% decrease in At) (Additional file 2:Table S11), indicating that the Dt genome of cotton may have experienced stronger artificial selection than the At subgenome, which is consistent with the previous conclusion based on whole-genome resequencing [28]. These results suggest that artificially selected genes played a major role in the decrease of gene expression diversity during domestication, but the expression diversity of non-selected genes was also affected during domestication.

Furthermore, besides the XP-CLR methods used above, we also identified candidate selective sweeps in rice based on two other methods, namely, population differentiation (Fst) [43] and the ratio of genetic diversity (π_wild_/π_dome_) [44] between the wild and domestic species, to explore whether the methods used to identify the candidate selective sweeps affected the pattern found in the CSGS. All the CSGS genes identified with the three different methods showed a higher degree of decreased expression diversity than those in the WGGS (Table 2, Additional file 1: Figure S7), indicating that the method did not have much effect on the observed pattern in the CSGS.

## Discussion

In 2012, using array hybridization, Hufford et al. observed decreased variation in the gene expression of candidate selected genes of domestic and improved maize [34]. In 2014, Bellucci et al. used RNA sequencing and de novo transcriptome assembly to investigate the genetic and expression diversity of common bean and its wild related species and observed that the domestic common bean had lower genetic and gene expression diversity [30]. In addition, the ancestor of lettuce also showed higher expression diversity than each of the six horticultural subtypes [45]. These three pioneering reports led to the question of whether the decrease of gene expression diversity is a general pattern in all or most domestic species. In this study, we collected reference genomes as well as statistically sufficient transcriptome datasets (more than 4 replicates for each tissue) of 4 domestic crops and 3 domestic animals to exclude the problem of sampling and data bias. Our comprehensive analysis shows that domestication does generally reduce gene expression diversity in both domestic plants and different kinds of domestic animals including insects, birds and mammals.

Previous population genomics studies and analysis on gene variation diversity in this study revealed that all the seven domestic species experienced decrease of genetic diversity (Additional file 1: Table S12 - S13) compared to their wild relatives. The synchronous decrease of genetic diversity and gene expression diversity suggested that the reduction in expression diversity may have been a direct consequence of reduced genetic diversity during domestication. However, both bottleneck and selection can lead to a decreased genetic diversity. Therefore, it is necessary to determine which is the main force driving the decreased expression diversity. To discriminate these two forces, we further explored the relationships between the decreased percentages of genetic diversity and expression diversity in each gene and found that the decreased percentage of expression diversity had no linear relationship with the decreased percentage of genetic diversity (Additional file 1: Figure S8), suggesting that bottlenecks, which would reduce genetic diversity at the whole-genome level, may not be the major factor resulting in the decreased expression diversity.

Furthermore, we also observed that the artificially selected genes experienced a severer decrease of gene expression diversity than did the WGGS and the non-CSGS, indicating that domestication-related selection may have been the main driver of the reduced expression diversity. Previous studies hypothesized that loss of expression diversity may be due to the stabilization of *cis*-regulated expression [34], and it has been pointed out that almost half of the mutations affecting the domestic phenotypes were caused by the mutations located in *cis*-regulatory regions [12, 20]. Therefore, we further explored the effects of decreased genetic diversity in *cis*-regulatory elements on the reduced expression diversity and scrutinized the results obtained by one previous study in cotton [28]. We chose 843 one-to-one regulated enhancer and gene pairs (which means that one enhancer can regulate only one gene and that this gene can be regulated by only that enhancer) in cotton, and calculated the genetic diversity of enhancers and expression diversity of the corresponding regulated genes. Both the genetic diversity of enhancers and the expression diversity of genes were significantly decreased in cotton (Additional file 1: Figure S9a). The number of enhancer-gene pairs that exhibited a synchronous decrease of genetic diversity and expression diversity accounted for the largest portion (32.7%) (Additional file 1: Figure S9b). The second largest portion (25.7%) included the pairs in which the genetic diversity of the enhancer was unchanged but the expression diversity of the corresponding regulated gene was decreased. This kind of pairs may be affected by selected transcription factor genes because one such gene can interact with many loci and affect many genes’ expression [46]. All these results suggest that decrease of the genetic diversity of an enhancer was often accompanied with the decrease of expression diversity of the corresponding regulated gene in cotton, indicating that selection on *cis*-regulatory elements may be an important force resulting in the decrease of expression diversity. However, some enhancer-gene pairs did not show synchronous decrease, this group of genes may not be selected in the identified enhancer but in other regulatory and even upstream *trans-*factors.

Among the three tissues of maize, only the ears exhibited significantly decreased expression diversity in the WGGS and CSGS, and exhibited a higher degree of decreased expression diversity than did the stem and leaf in the CSGS (Table 2). This phenomenon may be because the ear, which is the most important tissue affecting crop yields, had been subject to stronger selection pressure than the stem and leaf during domestication, which also indicates that decreased expression diversity may have tissue-specific characteristics due to different selected traits. Intriguingly, we also found an important domestication genes—KN-1, a transcription factor that affected the development of the cob [46] and showed decreased expression diversity in the ears (maize: 0.239 < teosinte: 0.402), further supporting the idea that domestication-related selection in *cis*-regulatory regions may have been the driving force of the decreased expression diversity.

Domestication, which is an evolutionary process that alters wild species to meet human needs, is often accompanied by many morphological and physiological changes. During this process, humans usually offer wild species a more stable and suitable environment than the harsh and variable environments in which the species previously lived [47] to facilitate the species’ growth and reproduction for food or other demands. Over generations of selection, the domestic species gradually gains adaptations to the suitable domestication environment, even if the adaptations were deleterious in the wild, such as the loss of shattering which made harvesting easier for farmers but prevented the spreading of seeds in the wild [48], and the loss of resistance to salt [49]. Although only a few genes are under selection [11, 12], due to the complex interactions between genes [46] and hitch-hiking effect [50] of many linked genes, the few selected genes may affect many other genes’ expression and then change the pattern of whole-genome gene expression. The reduced expression diversity (Table 2) suggested that some genes had lost their variable expression profiles and thus might have lost their variable functions used to adapt to varied environments. Therefore, domestication might have lost variability in both genetic and gene expression level in order to enhance the human-preferred traits, and thereby in this sense domestication process may well fit the “less is more” model [25]. However, the loss of both genetic diversity and expression diversity may make the domestic species vulnerable to the harsh wild environment and decrease their plasticity and eventually lead to domestication syndrome.

Because the expression of genes is affected by many factors, the lower expression diversity in domestic species may also have been caused by suitable environments or the loss of some trans-factors. In addition, gene expression also showed spatiotemporal [20] and tissue-specific [51] characteristics. Therefore, more evidence is necessary to support the pattern of decreased expression diversity during domestication. With the availability of more transcriptome data for more domestic species and their wild relatives in the future, the decrease of gene expression diversity may be supported by more examples in different species and different tissues, and it will be possible to clarify the driving force of reduced expression diversity.

## Conclusions

In summary, our current study observed a global decreased gene expression diversity during domestication in addition to the decrease of genetic diversity. The global decrease of gene expression diversity may have wide and profound effects on the phenotypic and morphological changes of domestic species compared with wild species. Our results provide insights into the genetic mechanisms underlying artificial selection.

## Materials and methods

### Sampling, RNA isolation and sequencing

We collected the young panicles of the rice, the stem apical meristems of soybeans and the silk glands of silkworms at the same development stages to extract RNA. The tissues were frozen in the liquid nitrogen and used for isolating RNAs using TRIzol (Invitrogen, USA). We chose 500 bp fragments to construct the RNA library, quantified the libraries with quantitative PCR and finally sent to sequencing on Hiseq 2000 platform, generating 100 bp paired-end sequencing reads for rice and soybean, 125 bp paired-end sequencing reads for silkworm. Finally, a total of 40 rice samples including 20 cultivated rice (*Oryza sativa*) accessions and 20 wild accessions were collected from the Asian countries including China, India, Indonesia (Additional file 1: Table S1); 35 soybean samples including 10 wild soybean accessions, 14 landraces and 11 improved cultivars were collected from China, Japan, South of Korea, Russia, Canada and America (Additional file 1: Table S2); 8 silkworm samples including 4 wild accessions and 4 domesticated accessions were collected from China (Additional file 1: Table S3).

### Data collection

The collected transcriptome data includes the data from rice, soybean, maize, cotton, dog, chicken, silkworm. All the transcriptome data in the same domestic-wild pairs are from the same tissue at the same developmental stage. Among them, panicle of rice, shoot apical meristems of soybean and silk glands of silkworm were generated by us. Leaf of cotton including 40 domesticated accessions and 10 wild accessions [28], ear, leaf, stem of maize including 12 maize accessions and 17 teosinte accessions [29], brain of dog including 5 dog samples and 6 gray wolf samples [22], gastrocnemius of chicken including 5 domesticated accessions and 4 red junglefowl [21] were collected from the NCBI (The National Center for Biotechnology Information). Ultimately, the data contains 3 animals and 4 crops that are total seven pairs’ transcriptome data and both the domestic and wild species have more than 4 replicates for the following analysis.

### Data processing

The genomes and gene annotation files of domestic dog, domestic silkworm, wild chicken, cultivar rice, wild rice, cultivar cotton, cultivar soybean and cultivar maize were used as reference genome when reads mapping. Among them, the reference genomes of dog (CanFam3.1), maize (AGPv3.26), cultivar rice (IRGSP-1.0.26), wild rice (AWHD00000000.34), soybean (V1.0.27) were downloaded from the Ensembl database (http://ensemblgenomes.org/). The reference genome of cotton was downloaded from COTTONGEN database (*Gossypium hirsutum* 1.1, https://www.cottongen.org/) [52], and the reference genome of domestic silkworm was acquired from a previously published paper [35]. For chicken, we acquired the mapped read counts for each individual from the author and the reference genome of wild chicken was download from Ensembl in October 2008 [21]. To measure the expression level differences between the domestic and the wild species, the raw sequencing data downloaded from the NCBI SRA database were firstly changed from SRA format to fastq format with SRAtoolkit [53], and the reads were filtered with a custom Perl script which excludes the reads with more than 10% Ns and with more than 30% low-quality bases. Among them, the reads of the silkworm were trimmed the first two bases and the last two bases and the final length of reads in silkworm is 121 bp. Then RNA sequencing reads for each sample were mapped onto the corresponding reference genome using Bowtie 2.2.4 [54] and TopHat 2.0.12 [55]. After mapping, to ensure the comparison comparable, it is necessary to keep the domestic species and the wild species have the same number of samples especially when calculating the expression diversity because of the introduction of the concept of the variance (standard deviation). Therefore, for maize, soybean, cotton, chicken and dog, which have different number of samples in domestic species and the wild species (Table 1), we have chosen the samples which have more clean reads to keep the number of samples the same (Additional file 2: Table S8). In addition, to avoid the bias caused by the lower sequencing depth in the exonic regions, the raw reads of biological replicates of maize were merged together to improve the average mapping depth. The average mapping depths of the three tissues of maize pairs turned from 5× for each sample to 10× for each accession (Additional file 1: Table S8). Finally, 6 maize accessions and 6 teosinte accessions were used to the following analysis (Additional file 1: Table S7). Samtools 0.1.19 [56] was used to calculate the mapping depths for each base in exonic regions and the average mapping depth for exons in the whole genome is calculated as the average depth of the bases located in those exons.

### Gene expression analysis

For each pair, the transcriptome data belonging to the domestic or wild species were treated as biological replicates for each group and Cufflinks [55] was used to normalize and calculate the expression level by the fragments per kilobases per million reads (FPKMs) method. After that, FPKM thresholds, such as 0, 0.1, 0.5, 1, 5, were used to identify the number of expressed genes and compare the number of expressed genes between the domestic and wild species in different threshold.

### Expression diversity

After the reads were mapped to the corresponding reference genome by TopHat, the number of the reads mapped to each gene were counted by HTseq 0.6.0 [57] with the default parameters, we used an R package named DESeq [58] to calculate the expression level and normalize the expression level to reduce the bias due to different amplification during PCR. Each gene’s expression diversity, which is also named coefficient of variation (CV), was calculated as the ratio between the SD (standard deviation) and the mean of the expression values, separately for domestic and wild species. And Student’s *t*-test was used to test whether the expression diversity values in the domestic species are significantly lower than in the wild species in the WGGS, CSGS and non-CSGS. Finally, the expression diversity of each species was represented by the average value of the genes’ expression diversity. Considering that the SD and mean are easily affected by the number of samples, therefore we have chosen the samples which have more clean reads in the process of reads mapping to analyze the expression diversity (Additional file 2: Table S8).

### Genetic diversity

For the six domestic-wild pairs including dog, silkworm, rice, cotton and soybean, the transcriptome data used to calculate the expression diversity were also used to detect single nucleotide polymorphisms (SNPs). After raw reads were mapped to the reference genome with TopHat 2.0.12 [55], Picard tools (v1.119, https://broadinstitute.github.io/picard/) was used to remove the duplicated reads and the mpileup program in the SAMtools package [56] was used to call the raw SNPs. The raw SNPs were filtered based on the following criteria: (1) the SNPs for which the total mapping depth or SNP quality was less than 30 were excluded; (2) only the biallelic SNPs were retained and the allele frequency had to be more than 0.05; (3) the genotypes with fewer than 3 supported reads and a genotype quality of less than 20 were treated as missing. The SNPs with more than 20% missing genotypes were excluded. After exclusion, each gene’s genetic diversity was calculated based on Nei’s methods [44].

### SNP calling and selective sweeps identification in rice

To identify the candidate selective sweeps for rice, a total of 144 whole genome sequencing data which included 42 wild rice accessions from NCBI (PRJEB2829) and 102 cultivates accessions from the 3000 Rice Genomes Project [37] were collected. The reads after the quality control were mapped to the reference genome (IRGSP-1.0.26) using Burrows-Wheeler Aligner (bwa v0.7.12) [59]. Then the mapped reads were converted into bam format and marked duplicates to lower down the biases due to PCR amplification with Picard tools (v1.119, https://broadinstitute.github.io/picard/). After the program RealignerTargetCreator and IndelRealigner of the Genome Analysis Toolkit (GATK v3.5) [60] were used to realign the reads around the indels, SNPs calling used the GVCF mode with HaplotypeCaller in GATK to produce an intermediate GVCF (genomic VCF) file for each sample. The final GVCF file which was acquired by merging the intermediate GVCF files together was passed to GenotypeGVCFs to produce a set of joint-called SNP and indel calls. Finally, the SNPs were selected and filtered with SelectVariants and VariantFiltration separately with the recommended parameters in GATK. The SNPs which have more than 30% were missing genotypes were excluded.

After acquiring the genetic mutation profiles of rice, an updated cross-population composite likelihood ratio test (XP-CLR, updated version, acquired from the author) [61], which is based on allele frequencies and deals with missing genotypes with an EM algorithm, was used to identify the candidate selective sweeps. A comparison between the cultivated population and the wild population was used to validate the selective sweeps that took place during domestication. The average physical distance per centimorgan (cM) was 244 kb for rice [62], therefore, we used a 0.05 cM sliding window with a 200 bp step to scan the whole genome, and each window had a maximum 200 SNPs in rice. After scanning, the average scores in 100 kb sliding window with 10 kb steps in the genome were estimated for each region. The regions with the highest 5% of scores were regarded as candidate selected regions. Finally, the overlapping regions within the top 5% of scores were merged together and treated as one selective sweep region, and the genes located in or overlapping with the candidate selective sweeps according to the gene coordinates were regarded as candidate selected genes.

Furthermore, we also used two other methods, namely, population differentiation (Fst) [43] and the ratio of genetic diversity (π_wild_/π_dome_) [44] between the wild and domestic species, to detect the candidate selective sweep regions in rice. VCFtools (version 0.1.13) [63] was used to calculate the Fst between the wild and domesticated populations, and the genetic diversity of wild and domesticated populations. A 100 kb sliding window with 10 kb step in the genome was used. Then, the regions with an Fst value or genetic diversity ratio in the top 5% were treated as candidate selective sweep regions. Finally, the overlapping regions were merged, and the genes located in these regions were treated as candidate selected genes.

## Additional files

Additional file 1: **Figures S1-S9** and **Table S1** to **Table S7** and **Table S12** to **Table S13**. (DOCX 1736 kb)

Additional file 2 **Table S8.** provides the mapping information for the seven pairs. **Table S9.** provides information about the resequencing data for rice. **Table S10.** provides the candidate selected genes for rice. **Table S11.** presents the expression diversity of the seven pairs. (XLSX 149 kb)

## Abbreviations

CSGS: Candidate selected gene set
CV: Coefficient of variation
GATK: Genome analysis toolkit
NCBI: National center for biotechnology information
Non-CSGC: None candidate selected gene set
RNA: Ribonucleic acid
SNPs: Single nucleotide polymorphisms
WGGS: Whole genome gene set
