# Supplementary material for: Decrease of gene expression diversity during domestication of animals and plants
Source: BMC Evol Biol. 2019 Jan 11;19:19. doi: 10.1186/s12862-018-1340-9 (PMC6330456; doi:10.1186/s12862-018-1340-9)
Supplement: Supplementary file 1 — Figures S1-S9 and Table S1 to Table S7 and Table S12 to Table S13. (DOCX 1736 kb) [file 12862_2018_1340_MOESM1_ESM.docx]

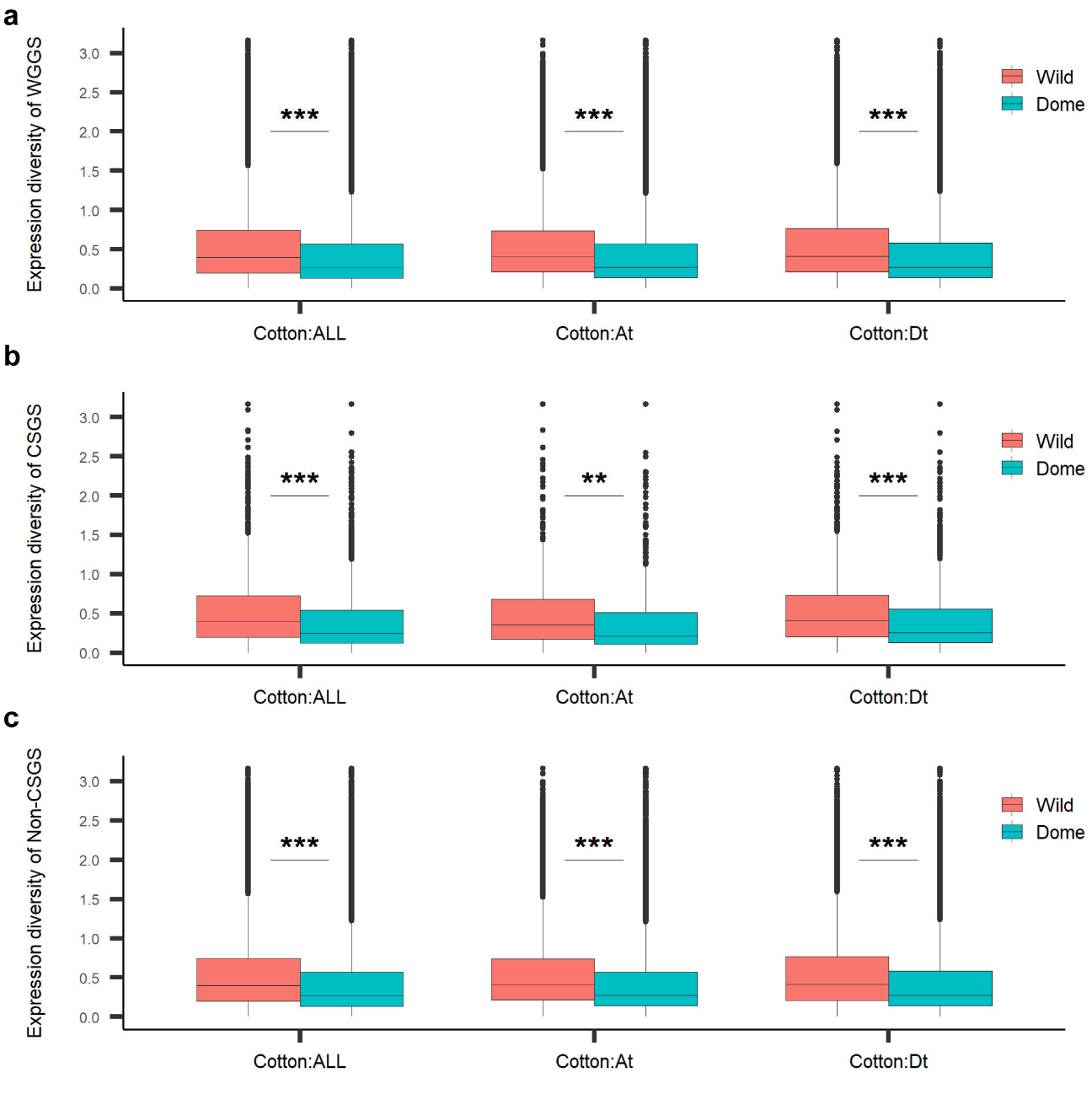


Figure S1. Gene expression diversity of the WGGS, CSGS and non-CSGS in cotton organism and two-subgenomes. The makers above the solid black lines are the *P*-value using Student’s *t*-test to test whether the expression diversity values in the domestic cotton are significantly lower than in the wild cotton. Tthe *P*-value less than 0.05, 0.01 and 0.001 were marked with *, ** and ***, separately. Both the whole genome and the two subgenomes showed significantly decreased expression diversity in the WGGS, CSGS and non-CSGS.


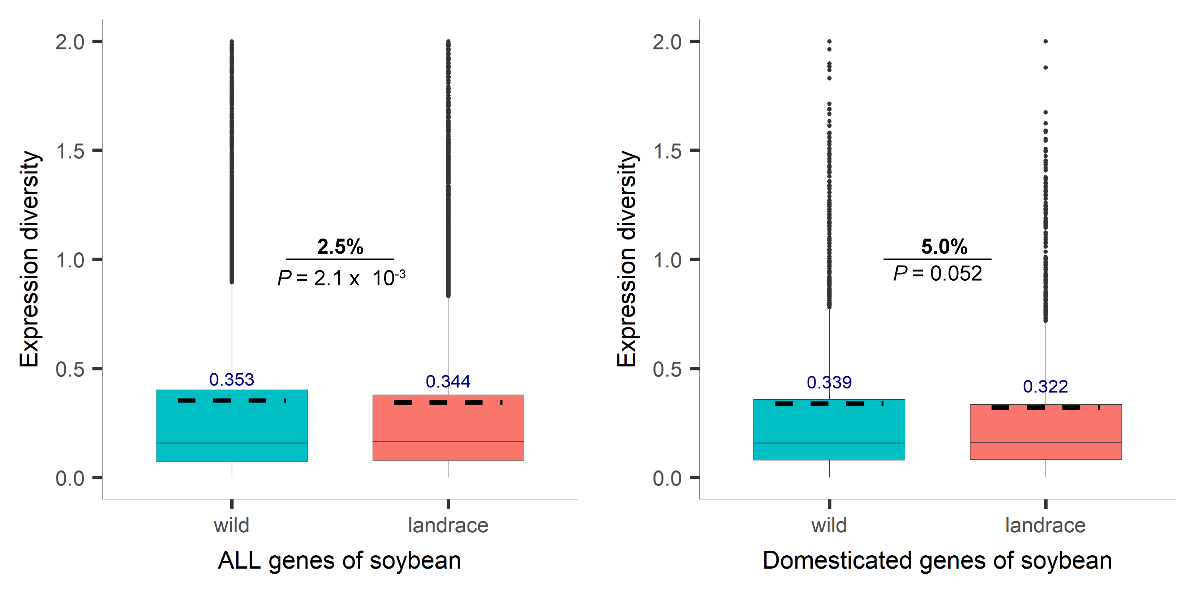


Figure S2. The expression diversity of soybeans when using four samples in the same group for both wild soybeans and landraces. (a) Expression diversity of the WGGS. (b) Expression diversity of the CSGS. Four landraces which belong to Group I-3 (Additional file 1: Table S2) and four wild soybeans which had relatively more clean reads (Additional file 1: Table S2) were used to compare the expression diversity between wild soybean and landraces. There is no expression diversity of improved cultivars because none of the groups in improved cultivars has more than three samples.

**
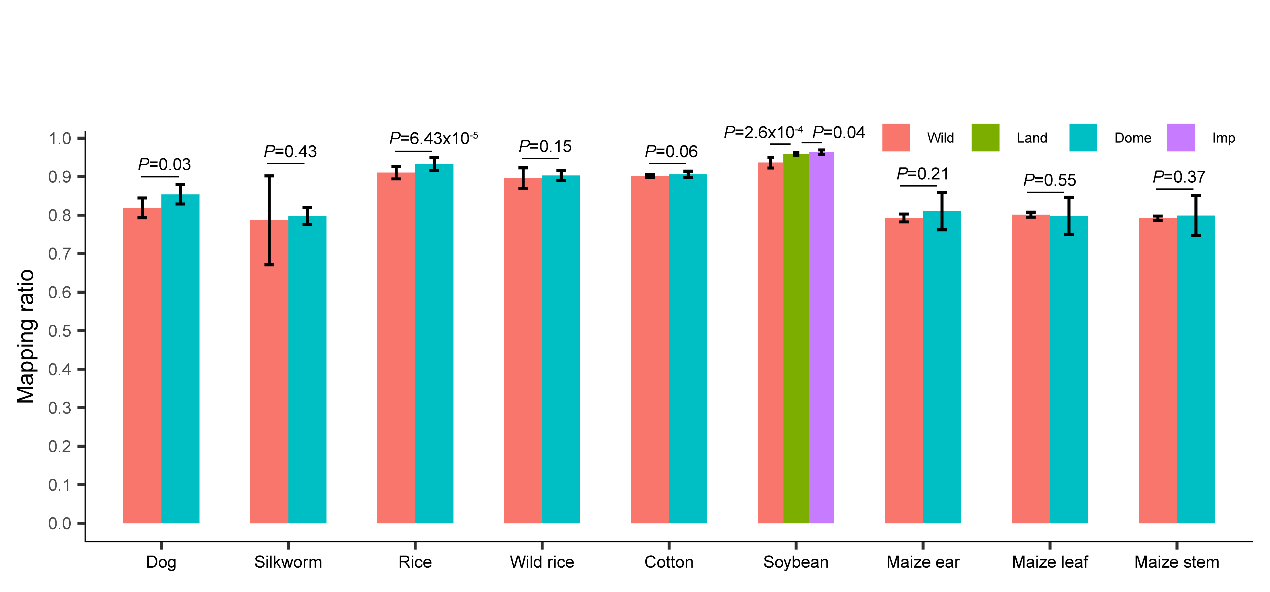
**

Figure S3. The distribution of mapping ratios for each domestic-wild pair. Rice means mapping with the genome of *Oryza japonica*. Wild rice means mapping with the genome of *Oryza nivara* (GCA_000576065.1). Student’s *t*-test was used to test whether the mapping ratio is significantly lower in wild species when using the genome of domestic species and the *P*-values were marked above the black lines. The mapping ratio of improved soybeans is also significantly higher (*P* = 5.65 🞨10^-5^) than the wild soybean.

**
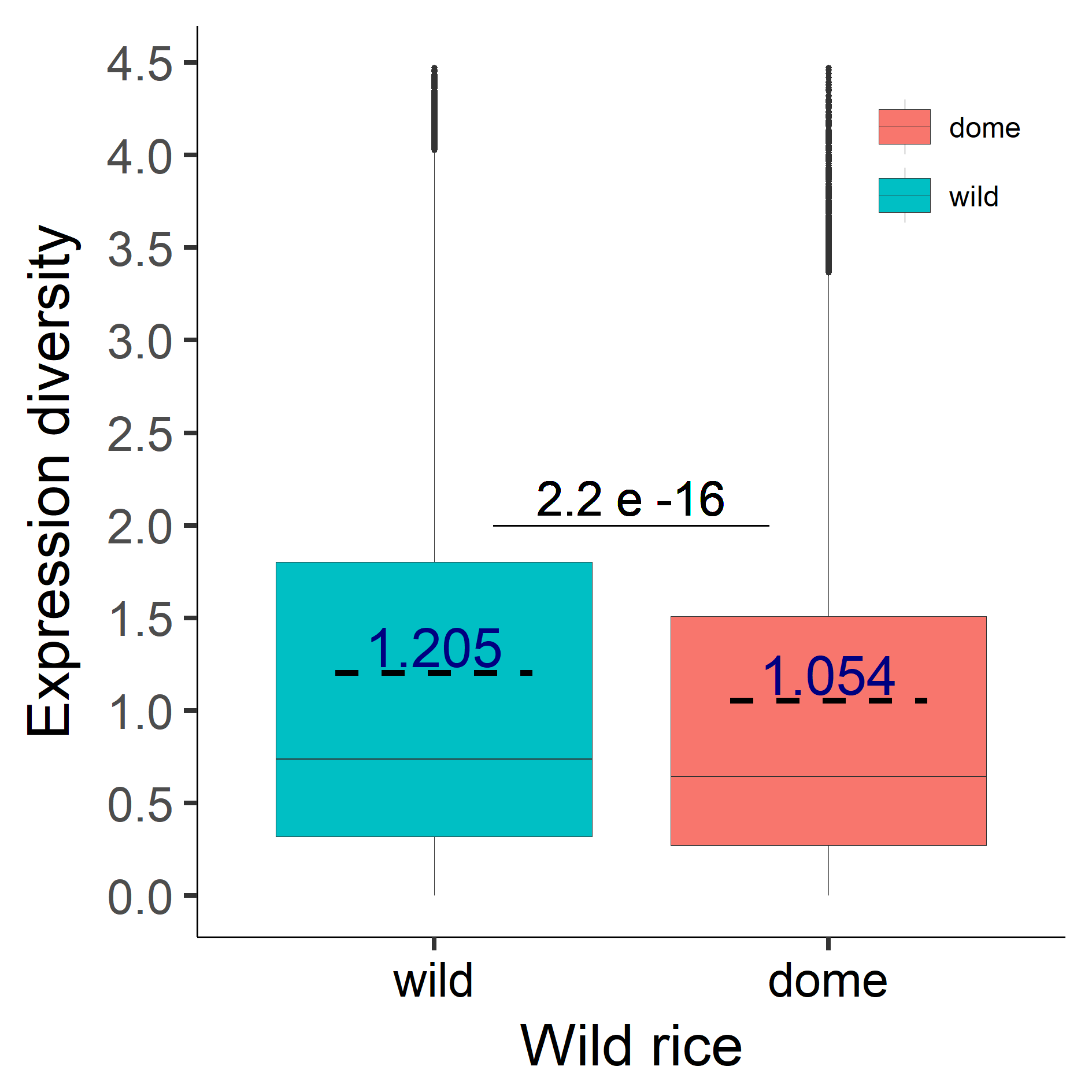
**

Figure S4. The gene expression diversity for rice organisms with the genome of *Oryza nivara* (GCA_000576065.1) as the reference genome. The black dashed lines represent the mean value of each gene’s expression diversity and the blue numbers above the dashed lines are the mean value of each gene’s expression diversity value. The numbers above the solid black lines are the *P*-value using Student’s *t*-test.


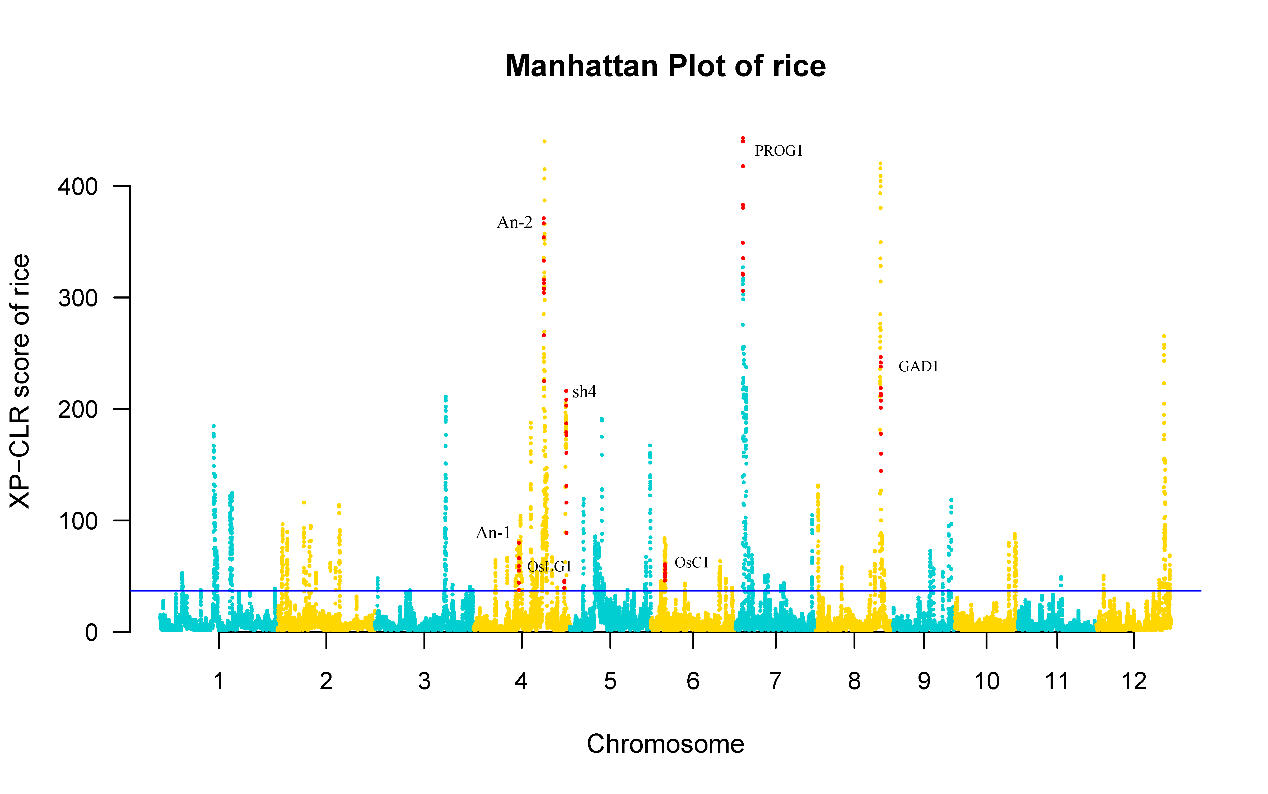


Figure S5. Genome-wide scanning and detecting artificially candidate selected regions in rice with XP-CLR. The scattered points above the blue line are the regions with the top 5% XP-CLR score. The blue line, which is 37.1, represents the threshold of artificially candidate selected regions and the red points represent the XP-CLR score of the seven well-characterized domesticated genes.


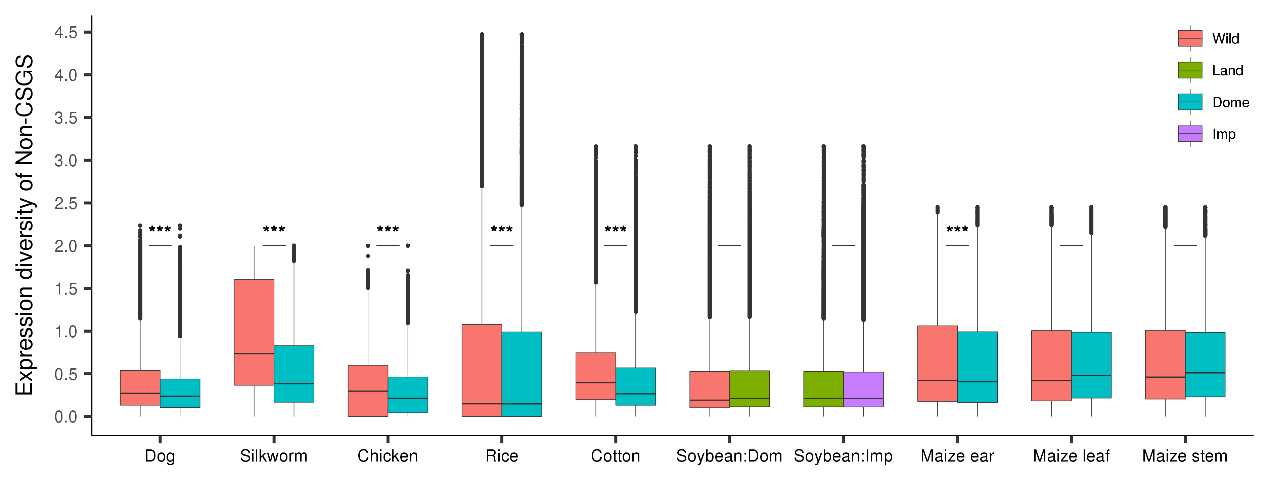


Figure S6. Gene expression diversity of the non-CSGS in the seven domestic organisms. From left to right, they represent the expression diversity for the non-CSGS genes in dog, silkworm, chicken, rice, cotton, ear, leaf and stem of maize, landrace and improved soybean, separately. *P*-value (Student’s *t*-test less) than 0.05, 0.01 and 0.001 were marked with *, ** and ***, separately.


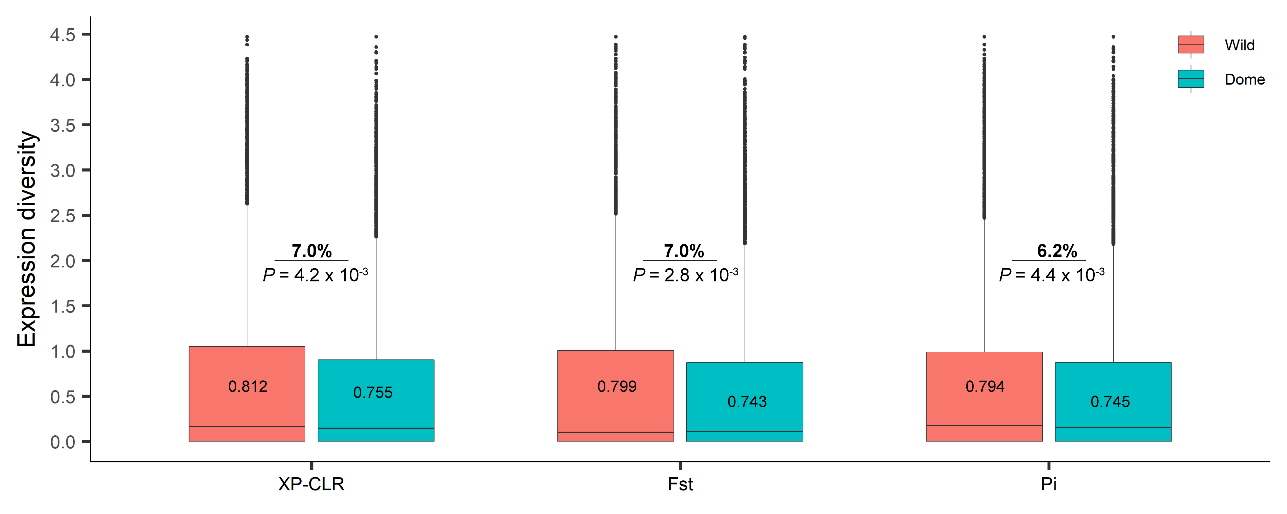


Figure S7. The expression diversity of the CSGS identified with XP-CLR, Fst and ratio of genetic diversity (π_wild_/π_dome_, Pi) in rice. The number in the box is the average expression diversity for domestic and wild rice. The number above the black line is the percentage of reduced expression diversity and the number under the black line is the *P*-value with Student’s *t*-test to test whether the expression diversity values in the domestic rice are significantly lower than in the wild rice.


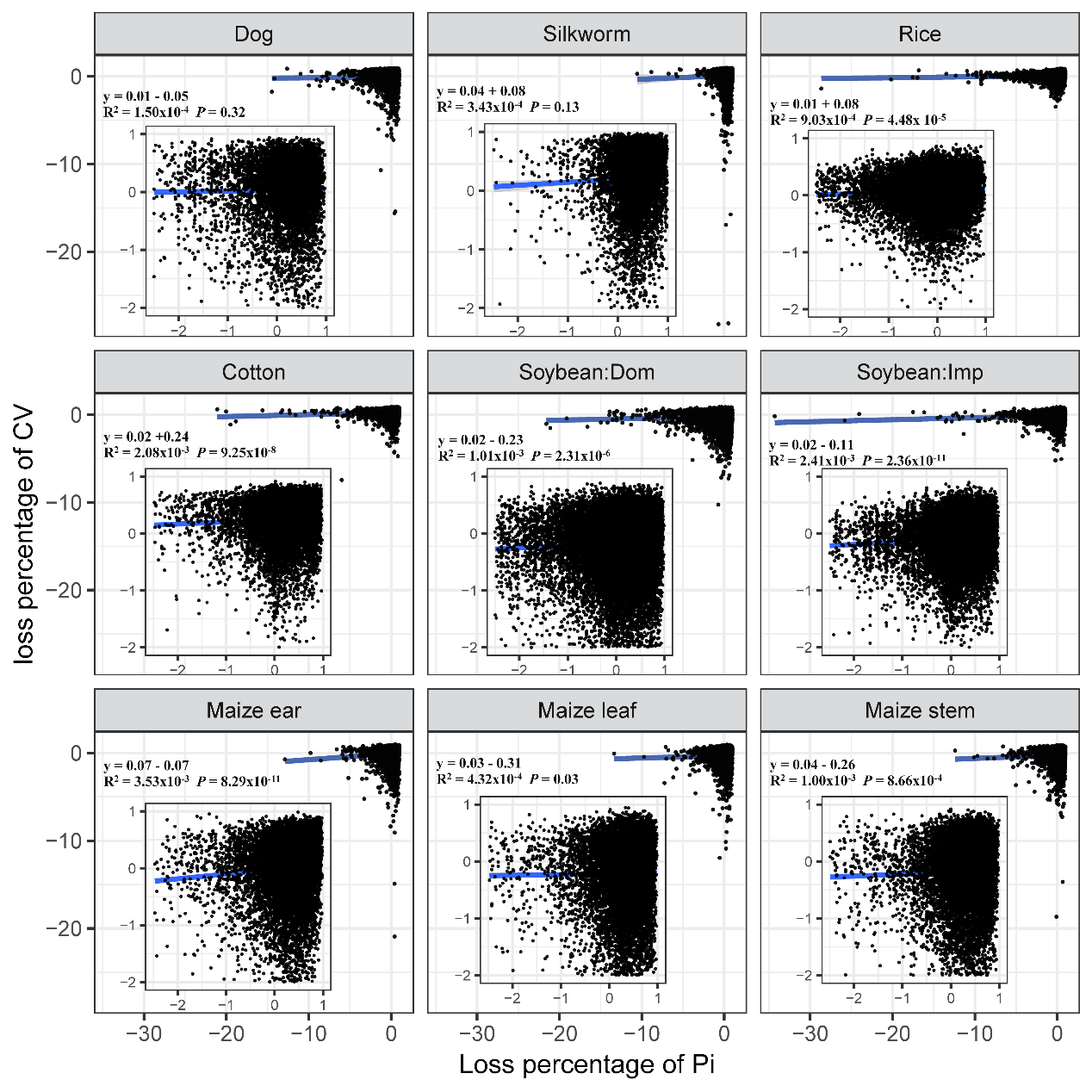


Figure S8. The relationships of the decreased percentages between the genetic diversity (Pi) and expression diversity (CV). The decreased percentage of genetic diversity of each gene was based on the transcriptome data used in this study. The closer the value of R^2^ is to 1, the more linear the relationship is. The little figures in the left bottom are a part of the large figures.


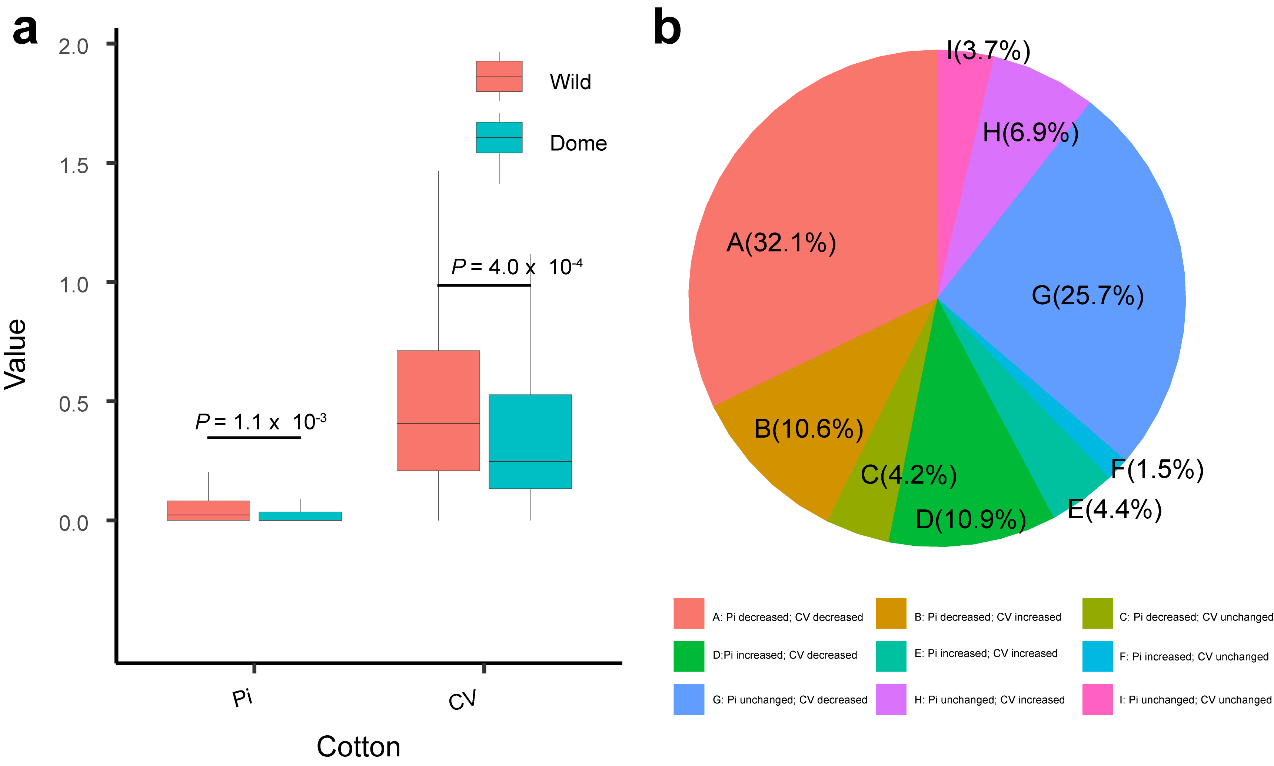


Figure S9. Genetic diversity (π, Pi) of enhancers and expression diversity (CV) of corresponding regulated genes in cotton. (a) the genetic diversity (Pi, ×10^-2^/bp) and expression diversity for the 843 one-to-one regulated enhancer and gene pairs (which means that one enhancer can regulate only one gene and that this gene can be regulated by only that enhancer) identified in cotton ([Wang, Tu et al. 2017](#_ENREF_1)). Student’s *t*-test was used to test whether the genetic diversity of enhancer and expression diversity of regulated genes were significantly decreased in the domestic cotton compared with its progenitors. (b) the statistics for the changes of genetic diversity of enhancers and expression diversity of the corresponding regulated genes.

Table S1. The summary sample information of rice.

| **Sample name** | **Accession number** | **Subgroup** | **Origin** |
| --- | --- | --- | --- |
| P01 | IRGC 9091 | *aromatic* | India |
| P02 | IRGC 12880 | *aromatic* | India |
| P03 | IRGC 53637 | *aromatic* | India |
| P04 | IRGC 6307 | *aus* | India |
| P05 | IRGC 8839 | *aus* | Bangladesh |
| P06 | IRGC 32399 | *temperate japonica* | Bhutan |
| P07 | IRGC 45195 | *aus* | India |
| P08 | IRGC 7755 | *indica* | Srilanka |
| P09 | IRGC 8240 | *indica* | Taiwan,China |
| P10 | IRGC 43369 | *indica* | Indonesia |
| P11 | IRGC 51250 | *indica* | China |
| P12 | IRGC 56036 | *indica* | Vietnan |
| P13 | IRGC 58930 | *indica* | Nepal |
| P14 | IRGC 1107 | *temperate japonica* | China |
| P15 | IRGC 55530 | *temperate japonica* | South Korea |
| P16 | IRGC 58286 | *temperate japonica* | Afghanistan |
| P17 | IRGC 25901 | *tropical japonica* | Bangladesh |
| P18 | IRGC 43325 | *tropical japonica* | Indonesia |
| P19 | IRGC 43397 | *tropical japonica* | Indonesia |
| P20 | IRGC 43675 | *tropical japonica* | Indonesia |
| P21 | Acc.80433 | *O.rufipogon* | India |
| P22 | Acc.80680 | *O.rufipogon* | India |
| P23 | Acc.80742 | *O.rufipogon* | Myanmar |
| P24 | Acc.81984 | *O.rufipogon* | Laos |
| P25 | Acc.81986 | *O.rufipogon* | Cambodia |
| P26 | Acc.103308 | *O.rufipogon* | Taiwan,China |
| P27 | Acc.105832 | *O.rufipogon* | Thailand |
| P28 | Acc.106133 | *O.rufipogon* | India |
| P29 | Acc.106138 | *O.rufipogon* | India |
| P30 | Acc.102159 | *O.rufipogon* | Thailand |
| P31 | Acc.80432 | *O.nivara* | India |
| P32 | Acc.80575 | *O.nivara* | India |
| P33 | Acc.80581 | *O.nivara* | India |
| P34 | Acc.80611 | *O.nivara* | India |
| P35 | Acc.80681 | *O.nivara* | India |
| P36 | Acc.80724 | *O.nivara* | Myanmar |
| P37 | Acc.100195 | *O.nivara* | Myanmar |
| P38 | Acc.100593 | *O.nivara* | Thailand |
| P39 | Acc.80696 | *O.nivara* | India |
| P40 | Acc.102167 | *O.nivara* | India |

Table S2. The summary sample information of soybean.

| **Field-id** | **Accession number** | **Group** | **Origin** | **Group in phylogenetic tree (Zhou et.al 2015)** |
| --- | --- | --- | --- | --- |
| TZX-250 | PI 562565 | *G.soja* | Cholla Puk, South Korea | Group-0 |
| TZX-264 | PI 407246 | *G.soja* | Kyongsang Puk, South Korea | Group-0 |
| TZX-262 | PI 407170 | *G.soja* | Kyonggi, South Korea | Group-0 |
| TZX-247 | PI 504286 | *G.soja* | Chungchong Puk, South Korea | Group-0 |
| TZX-241 | PI 366121 | *G.soja* | Fukushima, Japan | Group-0 |
| TZX-243 | PI 366123 | *G.soja* | Iwate, Japan | Group-0 |
| TZX-244 | PI 378692 | *G.soja* | Iwate, Japan | Group-0 |
| TZX-236 | PI 522216 | *G.soja* | Primorye, Russia | Group-0 |
| TZX-253 | PI 458536 | *G.soja* | Heilongjiang province, China | Group-0 |
| TZX-248 | PI 549046 | *G.soja* | Shanxi province, China | Group-0 |
| TZX-076 | PI 399043 | landrace | Cheju, South Korea | Group-I-1 |
| TZX-051 | PI 507355 | landrace | Hokkaido, Japan | Group-I-1 |
| TZX-089 | PI 594451 | landrace | Sichuan province, China | Group-I-2 |
| TZX-544 | Zhonghuang13 | improved cultivar | Beijing, China | Group-I-3 |
| TZX-081 | PI 437321 | landrace | Primorye, Russia | Group-I-3 |
| TZX-103 | PI 437679 | landrace | China | Group-I-3 |
| TZX-108 | PI 567503 | landrace | Hebei province, China | Group-I-3 |
| TZX-212 | PI 548402 | landrace | Beijing, China | Group-I-3 |
| TZX-082 | PI 437654 | landrace | unknown, China | Group-I-3 |
| TZX-515 | Ludou11 | improved cultivar | Shandong province, China | Group-II-1 |
| TZX-050 | PI 467343 | landrace | Jilin province, China | Group-II-1 |
| TZX-071 | PI 603336 | landrace | Heilongjiang province, China | Group-II-1 |
| TZX-176 | PI 548573 | improved cultivar | Ontario, Canada | Group-II-2 |
| TZX-191 | PI 548638 | improved cultivar | Ontario, Canada | Group-II-2 |
| TZX-171 | PI 533655 | improved cultivar | Illinois, America | Group-II-2 |
| TZX-001 | PI 547409 | improved cultivar | Illinois, America | Group-II-3 |
| TZX-433 | Jinda73 | improved cultivar | Shanxi province, China | Group-II-3 |
| TZX-172 | PI 540552 | improved cultivar | Ohio, America | Group-II-4 |
| TZX-429 | Jinda53 | improved cultivar | Shanxi province, China |  |
| TZX-616 | - | improved cultivar | China |  |
| TZX-315 | Williams 82 | improved cultivar | - |  |
| TZX-438 | - | landrace | Shanxi province, China |  |
| TZX-358 | - | landrace | Beijing, China |  |
| TZX-131 | PI 283327 | landrace | - |  |
| TZX-359 | - | landrace | - |  |

Table S3. The summary sample information of silkworm.

| **Sample name** | **Group** | **Origin** |
| --- | --- | --- |
|  |  |  |
| t1 | *Bombyx mori* (trimolter) | Domestic breed |
| t2 | *Bombyx mori* (trimolter) | Domestic breed |
| t3 | *Bombyx mori* (trimolter) | Domestic breed |
| t4 | *Bombyx mori* (trimolter) | Domestic breed |
| w1 | *Bombyx mandarina* | Wenling, China |
| w2 | *Bombyx mandarina* | Sichuan, China |
| w3 | *Bombyx mandarina* | Yunnan, China |
| w4 | *Bombyx mandarina* | Unknown |

Table S4. Transcriptome filtering information of rice.

| **Sample name** | **Species** |  | **Raw data** | |  | **Clean data** | | |
| --- | --- | --- | --- | --- | --- | --- | --- | --- |
|  |  | **Reads length(bp)** | **Raw**  **reads** | **Raw bases(Gb)** |  | **Reads length(bp)** | **Clean**  **reads** | **Clean bases(Gb)** |
| P01 | Rice | 100 | 74,130,399 | 14.826 |  | 100 | 66,955,946 | 13.391 |
| P02 | Rice | 100 | 11,378,682 | 2.276 |  | 100 | 10,657,496 | 2.131 |
| P03 | Rice | 100 | 17,102,063 | 3.420 |  | 100 | 15,286,694 | 3.057 |
| P04 | Rice | 100 | 27,206,273 | 5.441 |  | 100 | 24,375,614 | 4.875 |
| P05 | Rice | 100 | 31,763,487 | 6.353 |  | 100 | 28,669,901 | 5.734 |
| P06 | Rice | 100 | 24,581,386 | 4.916 |  | 100 | 23,106,979 | 4.621 |
| P07 | Rice | 100 | 51,123,364 | 10.225 |  | 100 | 45,456,944 | 9.091 |
| P08 | Rice | 100 | 46,983,460 | 9.397 |  | 100 | 42,574,847 | 8.515 |
| P09 | Rice | 100 | 42,178,034 | 8.436 |  | 100 | 36,619,013 | 7.324 |
| P10 | Rice | 100 | 38,976,315 | 7.795 |  | 100 | 36,430,643 | 7.286 |
| P11 | Rice | 100 | 17,611,114 | 3.522 |  | 100 | 16,151,886 | 3.230 |
| P12 | Rice | 100 | 41,385,787 | 8.277 |  | 100 | 38,105,929 | 7.621 |
| P13 | Rice | 100 | 88,625,114 | 17.725 |  | 100 | 82,667,516 | 16.534 |
| P14 | Rice | 100 | 63,491,008 | 12.698 |  | 100 | 56,290,761 | 11.258 |
| P15 | Rice | 100 | 52,621,499 | 10.524 |  | 100 | 48,180,088 | 9.636 |
| P16 | Rice | 100 | 58,750,403 | 11.750 |  | 100 | 53,710,156 | 10.742 |
| P17 | Rice | 100 | 28,696,349 | 5.739 |  | 100 | 26,372,265 | 5.274 |
| P18 | Rice | 100 | 28,174,661 | 5.635 |  | 100 | 24,970,400 | 4.994 |
| P19 | Rice | 100 | 32,736,415 | 6.547 |  | 100 | 28,275,192 | 5.655 |
| P20 | Rice | 100 | 37,400,276 | 7.480 |  | 100 | 34,040,711 | 6.808 |
| P21 | Rice | 100 | 8,094,372 | 1.619 |  | 100 | 6,611,915 | 1.322 |
| P22 | Rice | 100 | 33,972,508 | 6.795 |  | 100 | 31,570,434 | 6.314 |
| P23 | Rice | 100 | 35,932,457 | 7.186 |  | 100 | 33,173,431 | 6.635 |
| P24 | Rice | 100 | 43,683,528 | 8.737 |  | 100 | 39,340,201 | 7.868 |
| P25 | Rice | 100 | 17,897,812 | 3.580 |  | 100 | 15,942,796 | 3.189 |
| P26 | Rice | 100 | 25,080,325 | 5.016 |  | 100 | 23,346,165 | 4.669 |
| P27 | Rice | 100 | 44,886,574 | 8.977 |  | 100 | 42,547,065 | 8.509 |
| P28 | Rice | 100 | 34,970,194 | 6.994 |  | 100 | 30,642,653 | 6.129 |
| P29 | Rice | 100 | 31,351,472 | 6.270 |  | 100 | 28,996,400 | 5.799 |
| P30 | Rice | 100 | 22,998,831 | 4.600 |  | 100 | 21,589,974 | 4.318 |
| P31 | Rice | 100 | 19,716,494 | 3.943 |  | 100 | 18,156,708 | 3.631 |
| P32 | Rice | 100 | 7,398,472 | 1.480 |  | 100 | 6,799,511 | 1.360 |
| P33 | Rice | 100 | 68,647,508 | 13.730 |  | 100 | 57,097,414 | 11.419 |
| P34 | Rice | 100 | 114,768,600 | 22.954 |  | 100 | 103,090,887 | 20.618 |
| P35 | Rice | 100 | 18,748,738 | 3.750 |  | 100 | 16,566,511 | 3.313 |
| P36 | Rice | 100 | 34,077,626 | 6.816 |  | 100 | 31,526,288 | 6.305 |
| P37 | Rice | 100 | 58,714,955 | 11.743 |  | 100 | 51,749,729 | 10.350 |
| P38 | Rice | 100 | 28,663,303 | 5.733 |  | 100 | 26,096,378 | 5.219 |
| P39 | Rice | 100 | 29,871,581 | 5.974 |  | 100 | 27,548,140 | 5.510 |
| P40 | Rice | 100 | 31,430,062 | 6.286 |  | 100 | 28,783,097 | 5.757 |
| **Total** |  | **-** | **1,525,821,501** | **305.164** |  | **-** | **1,380,074,678** | **276.015** |

Table S5. Transcriptome filtering information of soybean.

| **Sample name** | **Species** | **Raw data** | | |  | **Clean data** | | |
| --- | --- | --- | --- | --- | --- | --- | --- | --- |
|  |  | **Reads length(bp)** | **Raw reads** | **Raw bases(Gb)** |  | **Reads length(bp)** | **Clean**  **reads** | **Clean bases(Gb)** |
| TZX-001 | Soybean | 100 | 19,207,983 | 3.842 |  | 100 | 18,346,787 | 3.669 |
| TZX-050 | Soybean | 100 | 25,640,336 | 5.128 |  | 100 | 24,829,186 | 4.966 |
| TZX-051 | Soybean | 100 | 22,610,264 | 4.522 |  | 100 | 21,570,281 | 4.314 |
| TZX-071 | Soybean | 100 | 36,918,829 | 7.384 |  | 100 | 35,486,608 | 7.097 |
| TZX-076 | Soybean | 100 | 62,117,887 | 12.424 |  | 100 | 59,352,603 | 11.871 |
| TZX-081 | Soybean | 100 | 35,109,755 | 7.022 |  | 100 | 33,504,938 | 6.701 |
| TZX-082 | Soybean | 100 | 32,819,590 | 6.564 |  | 100 | 31,064,704 | 6.213 |
| TZX-089 | Soybean | 100 | 17,879,257 | 3.576 |  | 100 | 16,975,531 | 3.395 |
| TZX-103 | Soybean | 100 | 9,532,907 | 1.907 |  | 100 | 9,250,921 | 1.850 |
| TZX-108 | Soybean | 100 | 11,504,549 | 2.301 |  | 100 | 11,131,904 | 2.226 |
| TZX-131 | Soybean | 100 | 39,964,287 | 7.993 |  | 100 | 38,139,291 | 7.628 |
| TZX-171 | Soybean | 100 | 15,440,529 | 3.088 |  | 100 | 14,902,978 | 2.981 |
| TZX-172 | Soybean | 100 | 29,363,766 | 5.873 |  | 100 | 28,485,682 | 5.697 |
| TZX-176 | Soybean | 100 | 21,975,038 | 4.395 |  | 100 | 13,179,769 | 2.636 |
| TZX-191 | Soybean | 100 | 22,721,512 | 4.544 |  | 100 | 21,849,027 | 4.370 |
| TZX-212 | Soybean | 100 | 26,628,046 | 5.326 |  | 100 | 25,283,405 | 5.057 |
| TZX-236 | Soybean | 100 | 19,593,055 | 3.919 |  | 100 | 18,739,955 | 3.748 |
| TZX-241 | Soybean | 100 | 20,267,521 | 4.054 |  | 100 | 19,206,434 | 3.841 |
| TZX-243 | Soybean | 100 | 21,235,349 | 4.247 |  | 100 | 20,552,331 | 4.110 |
| TZX-244 | Soybean | 100 | 21,041,748 | 4.208 |  | 100 | 20,204,176 | 4.041 |
| TZX-247 | Soybean | 100 | 29,986,750 | 5.997 |  | 100 | 28,241,166 | 5.648 |
| TZX-248 | Soybean | 100 | 21,712,836 | 4.343 |  | 100 | 20,648,887 | 4.130 |
| TZX-250 | Soybean | 100 | 16,970,635 | 3.394 |  | 100 | 16,128,118 | 3.226 |
| TZX-253 | Soybean | 100 | 23,035,076 | 4.607 |  | 100 | 22,090,549 | 4.418 |
| TZX-262 | Soybean | 100 | 32,996,270 | 6.599 |  | 100 | 31,587,408 | 6.317 |
| TZX-264 | Soybean | 100 | 31,242,840 | 6.249 |  | 100 | 28,774,855 | 5.755 |
| TZX-315 | Soybean | 100 | 24,084,073 | 4.817 |  | 100 | 23,031,464 | 4.606 |
| TZX-358 | Soybean | 100 | 33,493,980 | 6.699 |  | 100 | 31,865,233 | 6.373 |
| TZX-359 | Soybean | 100 | 18,638,658 | 3.728 |  | 100 | 17,751,255 | 3.550 |
| TZX-429 | Soybean | 100 | 20,423,830 | 4.085 |  | 100 | 18,900,803 | 3.780 |
| TZX-433 | Soybean | 100 | 36,709,886 | 7.342 |  | 100 | 34,065,263 | 6.813 |
| TZX-438 | Soybean | 100 | 34,738,980 | 6.948 |  | 100 | 32,857,324 | 6.571 |
| TZX-515 | Soybean | 100 | 22,456,783 | 4.491 |  | 100 | 21,453,068 | 4.291 |
| TZX-544 | Soybean | 100 | 31,866,238 | 6.373 |  | 100 | 29,608,185 | 5.922 |
| TZX-616 | Soybean | 100 | 30,325,696 | 6.065 |  | 100 | 28,679,416 | 5.736 |
| **Total** |  | **-** | **920,254,739** | **184.051** |  | **-** | **867,739,505** | **173.548** |

Table S6. Transcriptome filtering information of silkworm.

| **Sample name** | **Species** | **Raw data** | | |  | **Clean data** | | |
| --- | --- | --- | --- | --- | --- | --- | --- | --- |
|  |  | **Reads length(bp)** | **Raw**  **reads** | **Raw bases(Gb)** |  | **Reads length(bp)** | **Clean**  **reads** | **Clean bases(Gb)** |
| t1 | Sikworm | 125 | 27,838,003 | 6.960 |  | 121 | 26,867,168 | 6.502 |
| t2 | Sikworm | 125 | 30,537,293 | 7.634 |  | 121 | 29,706,661 | 7.189 |
| t3 | Sikworm | 125 | 30,695,052 | 7.674 |  | 121 | 30,202,803 | 7.309 |
| t4 | Sikworm | 125 | 25,105,463 | 6.276 |  | 121 | 24,789,438 | 5.999 |
| w1 | Sikworm | 125 | 31,884,735 | 7.971 |  | 121 | 31,442,000 | 7.609 |
| w2 | Sikworm | 125 | 27,823,452 | 6.956 |  | 121 | 27,509,479 | 6.657 |
| w3 | Sikworm | 125 | 28,990,919 | 7.248 |  | 121 | 28,534,127 | 6.905 |
| w4 | Sikworm | 125 | 25,265,607 | 6.316 |  | 121 | 24,894,384 | 6.024 |
| **Total** |  | **-** | **228,140,524** | **57.035** |  | **-** | **223,946,060** | **54.195** |

Table S7. The average mapping depths in exonic regions and the number of expressed genes for each species.

| **Species** | **Type** | **Sample size** | **Average mapping depth** | **Gene number** | **Number of expressed genes** | | | | |
| --- | --- | --- | --- | --- | --- | --- | --- | --- | --- |
|  |  |  |  |  | **FPKM>0** | **FPKM>0.1** | **FPKM>0.5** | **FPKM>1** | **FPKM>5** |
| Rice | Dome | 20 | 75.3 | 91080 | 38002 | 33831 | 30001 | 27712 | 19881 |
|  | Wild | 20 | 60.2 |  | 38052 | 34670 | 30567 | 28140 | 20077 |
| Soybean | Improved | 10 | 31.2 | 54174 | 46776 | 40512 | 35179 | 32133 | 20777 |
|  | Landrace | 10 | 43.4 |  | 47576 | 40797 | 35344 | 32240 | 20850 |
|  | Wild | 10 | 28.7 |  | 47208 | 40891 | 35512 | 32288 | 20710 |
| Maize ear | Dome | 6 | 10.3 | 39621 | 35891 | 31203 | 25268 | 22670 | 16437 |
|  | Wild | 6 | 10.2 |  | 35269 | 30306 | 24725 | 22398 | 16369 |
| Maize leaf | Dome | 6 | 10 | 39621 | 35733 | 31169 | 25173 | 22527 | 15475 |
|  | Wild | 6 | 11.5 |  | 34956 | 29338 | 23865 | 21536 | 15056 |
| Maize stem | Dome | 6 | 9.4 | 39621 | 36014 | 32379 | 26545 | 23885 | 16495 |
|  | Wild | 6 | 10.2 |  | 35457 | 31050 | 25856 | 23388 | 16272 |
| Cotton | Dome | 10 | 44.7 | 70478 | 60381 | 53859 | 47829 | 44078 | 29956 |
|  | wild | 10 | 40.1 |  | 58204 | 54208 | 48686 | 45059 | 30686 |
| Silkworm | Dome | 4 | 106.7 | 15665 | 12045 | 10436 | 9111 | 8431 | 6194 |
|  | Wild | 4 | 102.2 |  | 13290 | 10868 | 9356 | 8577 | 6075 |
| Dog | Dome | 5 | 15.3 | 24580 | 18889 | 17204 | 15196 | 14169 | 10548 |
|  | Wild | 5 | 16.4 |  | 19045 | 17355 | 15256 | 14185 | 10603 |
| Chicken | Dome | 4 | - | 17858 | 15059 | 13514 | 11013 | 9773 | 5874 |
|  | Wild | 4 | - |  | 14768 | 13380 | 10812 | 9494 | 5475 |

Average mapping depth is the average mapping depth for exons in the whole genome, which only take the exonic region into consideration. The average mapping depth for the gastrocnemius of chicken is unknown because only the reads uniquely mapped to each gene were acquired from the author without the raw reads data. The number of the genes for each organism was acquired from the annotation file. Among them, the genes of rice include 55401 non-coding RNA, thus the number of the genes in rice is much more than others. To keep the number of samples consistent in maize, soybean, cotton, chicken and dog and their wild progenitors, the top few samples which relatively have more cleaned reads was chosen to calculate the expression level (FPKMs) (Table S8). Different thresholds including 0, 0.1, 0.5, 1, 5, were tried to identify the number of expressed genes.

Table S12. Comparison of the average genetic diversity of genes between domestic species and wild species based on the transcriptome data used in this study.

| **Organisms** | **Population** | **Sample number** | **Average Genetic diversity of genes(π)** | **Decrease percentage of genetic diversity** |
| --- | --- | --- | --- | --- |
| Dog | Wild | 5 | 5.88E-05 |  |
|  | Dome | 5 | 3.20E-05 | 45.62% |
| Silkworm | Wild | 4 | 6.73E-04 |  |
|  | Dome | 4 | 3.98E-04 | 40.87% |
| Rice | Wild | 20 | 1.80E-04 |  |
|  | Dome | 20 | 1.68E-04 | 6.68% |
| Soybean | Wild | 10 | 2.15E-04 |  |
|  | Landrace | 10 | 1.61E-04 | 24.78% |
|  | Improved | 10 | 1.17E-04 | 27.81% |
| Maize ear | Wild | 6 | 2.34E-04 |  |
|  | Dome | 6 | 1.34E-04 | 42.74% |
| Maize leaf | Wild | 6 | 2.25E-04 |  |
|  | Dome | 6 | 1.26E-04 | 43.71% |
| Maize stem | Wild | 6 | 2.15E-04 |  |
|  | Dome | 6 | 1.24E-04 | 42.41% |
| Cotton | Wild | 10 | 1.39E-04 |  |
|  | Dome | 10 | 8.08E-05 | 41.99% |
| Cotton At | Wild | 10 | 1.40E-04 |  |
|  | Dome | 10 | 8.08E-05 | 42.16% |
| Cotton Dt | Wild | 10 | 1.47E-04 |  |
|  | Dome | 10 | 8.45E-05 | 42.58% |

Dome, domestic population; Wild, wild population. Decrease percentage of genetic diversity is equal to 1-(π_dome_/π_wild_).

Table S13. Comparison of the genetic diversity between domestic species and wild species according to previously published researches.

| **Organisms** | **Population** | **Sample number** | **Genetic diversity(****π)** | **Decrease percentage of genetic diversity** | **Publication** |
| --- | --- | --- | --- | --- | --- |
| Dog | Wild | 4 | 0.00141 |  | Wang *et al*., 2013 |
|  | Dome | 4 | 0.00073 | 48.23% |  |
| Silkworm | Wild | 19 | 0.0129 |  | Xiang *et al*., 2018 |
|  | Dome | 19 | 0.0096 | 25.5% |  |
| Chicken | Wild | 2 | 0.000407 |  | Mugal *et al*.,2013 |
|  | Dome | 4 | 0.00037 | 9.09% |  |
| Rice | Wild | 446 | 0.003 |  | Huang *et al*., 2012 |
|  | Dome | 1083 | 0.0024 | 20.00% |  |
| Soybean | Wild | 62 | 0.00294 |  | Zhou *et al*., 2015 |
|  | Landrace | 130 | 0.0014 | 52.38% |  |
|  | Improved | 110 | 0.00105 | 25.00% |  |
| Maize | Wild | 17 | 0.0059 |  | Hufford *et al*., 2012 |
|  | Dome | 23 | 0.0048 | 18.64% |  |
| Cotton | Wild | 30 | 0.00132 |  | Wang *et al*., 2017 |
|  | Dome | 267 | 0.00067 | 49.24% |  |
| Cotton.At | Wild | 30 | 0.00136 |  |  |
|  | Dome | 267 | 0.00072 | 47.06% |  |
| Cotton.Dt | Wild | 30 | 0.00125 |  |  |
|  | Dome | 267 | 0.00056 | 55.20% |  |

Dome, domestic population; Wild, wild population. Decrease percentage of genetic diversity is equal to 1-(π_dome_/π_wild_).
